# Subtyping β-lactamase-producing *Escherichia coli* strains isolated from patients with UTI by MLVA and PFGE methods 

**DOI:** 10.22038/ijbms.2021.49790.11372

**Published:** 2021-04

**Authors:** Alireza Dolatyar Dehkharghani, Setareh Haghighat, Marjan Rahnamaye Farzami, Masoumeh Douraghi, Mohammad Rahbar

**Affiliations:** 1 Department of Microbiology, Faculty of Advanced Sciences and Technology, Tehran Medical Sciences, Islamic Azad University, Tehran, Iran; 2 Department of Microbiology, Research Center of Reference Health Laboratory, Ministry of Health and Medical Education, Tehran, Iran; 3 Division of Microbiology, Department of Pathobiology, School of Public Health, Tehran University of Medical Sciences, Tehran, Iran

**Keywords:** Beta-lactamase, Escherichia coli, Molecular typing, Pulsed-field gel – electrophoresis, VNTR

## Abstract

**Objective(s):**

Strain subtyping is an important epidemiological tool to trace contamination, determine clonal relationships between different strains, and the cause of outbreaks. Current subtyping methods, however, yield less than optimal subtype discrimination. Pulsed-field gel electrophoresis is the gold standard method for *Escherichia coli* and Multiple-Locus Variable-number tandem repeat Analysis is a rapid PCR-based method. The purpose of this study was to evaluate MLVA and PFGE methods for subtyping β -lactamase-producing *E. coli* strains isolated from urinary tract infections.

**Materials and Methods:**

Overall, 230 *E. coli* isolates from patients with urinary tract infections were examined for antimicrobial susceptibility testing. 10-loci and 7-loci MLVA and PFGE methods were used for molecular typing of β -lactamase-producing *E. coli* isolates.

**Results:**

Out of 230 isolates, 130 (56.5%) β -lactamase-producing *E. coli* isolates were found in this study. The diversity indices of the VNTR loci showed an average diversity of 0.48 and 0.54 for 7-loci and 10-loci MLVA, respectively. The discriminatory power of PFGE showed a value of 0.87. The discordance between the methods was high.

**Conclusion:**

Our study showed that PFGE is more discriminatory than MVLA. MLVA is a PCR- based method and can generate unmistakable data, in contrast to PFGE. Optimization of polymorphic VNTR is essential to improve the discriminatory power of MLVA based on geographical region.

## Introduction

*Escherichia coli *is the most common bacteria causing urinary tract infections (1)*. E. coli* accounts for more than 85% of all urinary tract infections (2). One hundred and fifty million people are affected by urinary tract infections each year worldwide (3). Females are affected by urinary tract infections (UTI) up to 30 times more than males and about 60% of them get a UTI at least once in their lives (3).

The extended-spectrum beta-lactamases (ESBLs) hydrolyze oxyiminocephalosporin, and monobactams are class A plasmid-mediated enzymes. They are inhibited by clavulanic acid *in vitro* (4, 5). ESBL-producing bacteria can cause resistance to penicillin, narrow and extended-spectrum cephalosporin, and aztreonam antibiotics. They can commonly show resistance to aminoglycosides, trimethoprim/sulfamethoxazole, and quinolones (6, 7).

AmpC β-lactamases are encoded chromosomally or can be plasmid-mediated. The plasmid-mediated AmpC β-lactamases can hydrolyze β-lactam antimicrobials except for cefepime and carbapenems. Detection of AmpC is clinically significant to manage the patients suffering from infections. However, there are no standard guidelines for the detection of AmpC mediated resistance in Gram-negative bacilli and so false results can be usually observed, especially by phenotypic methods (8). Healthcare-associated and community-acquired infections are considered as two important sources of *E. coli *infection (9).

The identification of sources of contamination and the detection of routes of transmission are performed by genetic clustering. This can allow us to detect and control the dissemination of infections more accurately. Different molecular typing techniques have been broadly utilized in molecular epidemiological investigations of bacterial infectious diseases to study the source and relationship between strains (10). Among molecular typing techniques, the pulsed-field gel electrophoresis (PFGE) as a “gold standard” method, the multiple-locus variable-number tandem repeat analysis (MLVA), and the rRNA gene fingerprinting (ribotyping) show good efficacy in examination of outbreaks and sporadic cases (11-13). PFGE is a powerful molecular typing method and is used most commonly for bacterial subtyping. In the PFGE method, restriction enzymes are used to cut the genome of bacteria and to generate a limited number of high-molecular-weight restriction fragments (14, 15). These fragments are then separated by gel electrophoresis with programmed variables in both direction and duration of the pulsed electric field. MLVA and ribotyping are the other two frequently used molecular typing techniques for bacteria (16, 17). In this study, we evaluated the PFGE and MLVA methods for subtyping of AmpC-producing *E. coli *strains as well-defined group, and additionally, the ability of PFGE and MLVA typing was assessed and compared.

## Materials and Methods

During the years 2017 and 2018, two hundred and thirty isolates of β-lactamase-producing *E. coli* were collected from hospitalized patients with UTI symptoms at Milad hospital (general hospital in Tehran, IRAN). Isolates were identified using standard biochemical tests, and all isolates were kept at -80 °C using tryptic soy broth plus 15% glycerol for further molecular studies. 

***Antimicrobial susceptibility testing***

The isolates identified as *E. coli* were examined by E-test (minimum inhibitory concentration: MIC) method according to the guidelines of clinical and laboratory standards institute (CLSI) (18). In this method, the following antibiotics were used: ceftazidime (30 μg), gentamicin (10 μg), amoxicillin- (30 μg), meropenem (10 μg), trimethoprim-sulfamethoxazole (1.25 μg), ciprofloxacin (5 μg), cefoxitin (30 μg), and piperacillin-tazobactam (100/10 μg)(Mast Diagnostics Ltd). 

***Detection of ESBL-positive isolates***

Screening for ESBL production was done using 30 μg ceftazidime disc (CAZ) by disk diffusion method according to CLSI guidelines (CLSI 2018). Isolates showing zone of inhibition < 22 mm were considered as non-susceptible to ceftazidime (CAZ), i.e., potential ESBL producer. A disk of ceftazidime (CAZ) (30 μg) and a combined disk of ceftazidime with clavulanic acid (CAC) (30/10 μg) were used as a confirmatory test for each ESBL-possible isolate. Both of the disks were placed on a Muller Hinton agar (MHA) plate and incubated overnight at 37 °C. A ≥ 5 mm increase in zone diameter for each gram negative bacilli was considered ESBL positive (18). 

***Detection*** ***of AmpC-positive isolates***

A screening test for the detection of AmpC-positive isolates was performed on *E. coli* strains isolated from urine specimen using cefoxitin(30 µg) disk. In the next step, suspected strains were confirmed by the AmpC detection set (MASTDISCS™ID, UK). Finally, the results of antimicrobial susceptibility testing (AST) and AmpC detection tests were recorded in WHONET software. 

***Multi-locus variable number of tandem repeat analysis (MLVA)***

The whole genome of *E. coli *was extracted from overnight cultures using the high* pure* PCR *template* preparation *kit* (Roche, Germany). *E. coli* MLVA was done using seven variable number tandem repeats (VNTRs) (CVN001, CVN002, CVN003, CVN004, CVN007, CVN014, and CVN015) as described by Lindstedt *et al* (19). Additionally, CVN016 and CVN017 and one regularly interspersed short palindromic repeat (CCR001) that have been proven to improve the discriminatory power of this MLVA, and 10-loci *E. coli* MLVA have been designed accordingly (20). The primers were listed in Table 1. Repeats were amplified using PCR and evaluated on 3% agarose. 100-bp and 20-bp ladders (Bio-Rad) were utilized as a DNA size marker for VNTRs.

VNTR repeat numbers for each locus were calculated by the following formula; ((NPS−OF)/RL, where PS= product size, OF=offset region, and RL=length of one repeat unit. CVN001: ((PS)−250)/39, CVN002: ((PS)−272)/18, CVN003: ((PS)−404)/15, CVN004: ((PS)−231)/ 15, CVN007: ((PS)−314)/18, CVN014: ((PS)−111)/6, CVN015: ((PS)−189)/6, CCR001: ((PS)−131)/59, CVN016: ((PS)−478)/6 and CVN017: ((PS)−202)/6. All results were rounded to the nearest number [13]. The offset region includes the region of sequence not containing repeat. 

***Pulsed field gel electrophoresis***

Urinary *E. coli* isolates from an overnight culture were used to prepare solid agarose plugs and then incubated 2 hr at 55 °C in lysis buffer by proteinase K. The plugs were washed by 55–56 °C sterile distilled water and TE buffer (10 mM Tris:1 mM EDTA, pH 8.0). A 2 mm slice of the plug was cut and digested 2 hr with 50 U of XbaI restriction enzyme according to the manufacturer’s instructions (Thermo Fisher Scientific, USA).

PFGE was performed with the CHEF Mapper XA (Bio-Rad Laboratories, Hercules, CA) system using a 1% pulsed-field certified agarose gel (Bio-Rad Laboratories, Hercules, CA) in 2.2 liters 0.5X tris-buffered EDTA running buffer (Thermo Fisher Scientific, USA). The electrophoretic conditions used were as follows: initial switch time, 2.16 sec; final switch time, 54.17 sec; run time, 21 hr; gradient, 6 V/cm; angle, 120°; temperature, 14 °C. *Salmonella enterica* serotype Braenderup H9812 was used as a standard at each work serie along with the *E. coli* isolates to be tested (21).

***Comparison of MLVA and PFGE profiles***

All PFGE and MLVA profiles were processed in BioNumerics 6.6 (Applied Maths, Sint-Martens-Latem, Belgium), using UPGMA (unweighted pair group method with arithmetic mean) algorithm. Dice coefficient and Pearson correlation coefficient were used for PFGE and MLVA, respectively. This software was used for investigation of the diversity (Simpson’s index of diversity).

## Results

A total of 230 isolates were collected during the years 2017 and 2018. We are aiming to use these methods for outbreak detection and infection control in a hospital setting, so, the date and the location are the key pieces of information to help in the interpretation of the data. In this study, we only had access to the specimen collection wards.

***Typing of β -lactamase-producing E. coli isolates by PFGE***

**Figure 1:**
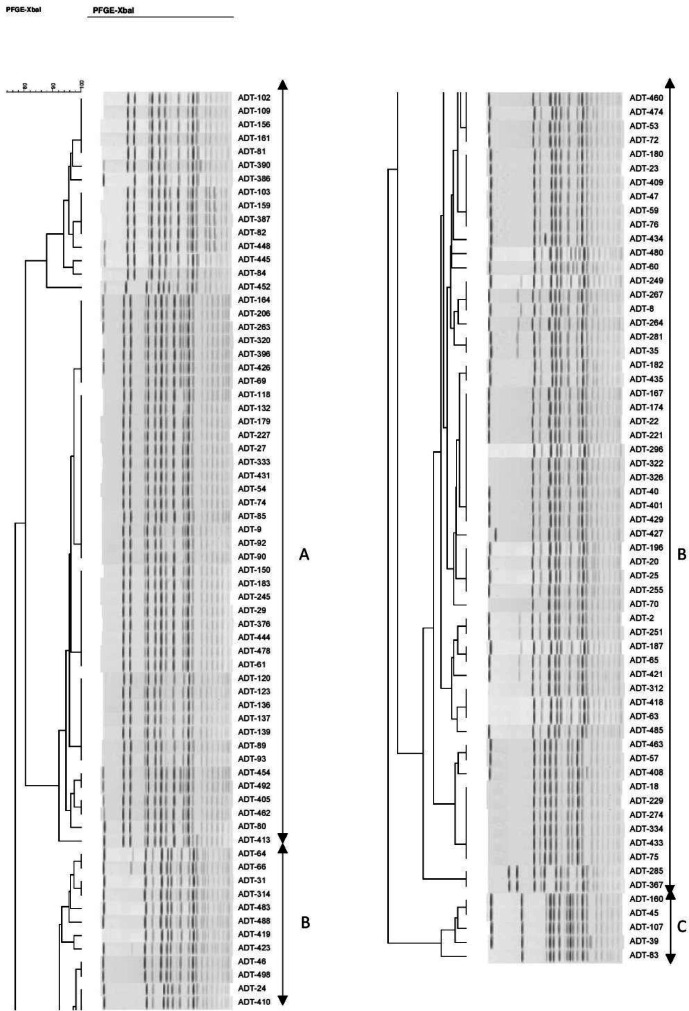
Clustering results of β -lactamase-producing *E. coli *using PFGE

A dendrogram was created by BioNumerics software ver. 6.6 and 49 *pulso*types formed 3 clusters based on their genetic similarity (cutoff *of *80%*)* and designated A to C. (Figure 1). The PFGE pattern of the β -lactamase producers and the comparison between the patterns were illustrated in Table 2.

***Typing of β -lactamase-producing E. coli isolates by MLVA***

**Figure 2:**
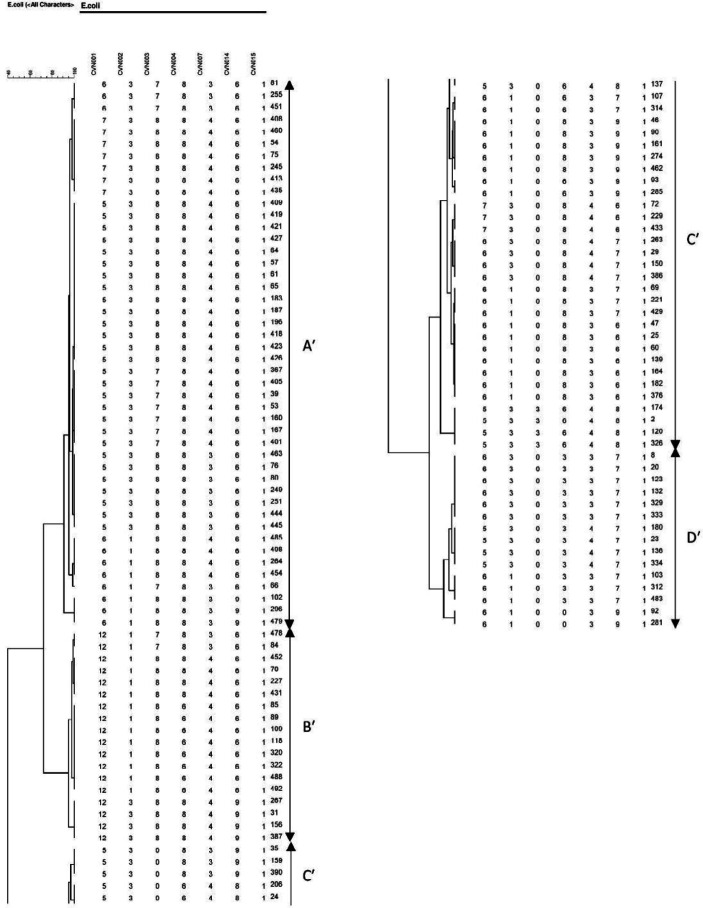
Clustering results of β -lactamase-producing *E. coli *using 7-loci MLVA

A dendrogram based on 10-loci and 7-loci MLVA profiles was created by BioNumerics software ver. 6.6. In this study, 7-loci MLVA-typing divided the β -lactamase-producing *E. coli* isolates into 26 distinct genotypes and 4 clusters designated A′ to D′ (Figure 2). The MLVA pattern of the β-lactamase producers and the comparison between the patterns were illustrated in Table 2.

**Figure 3:**
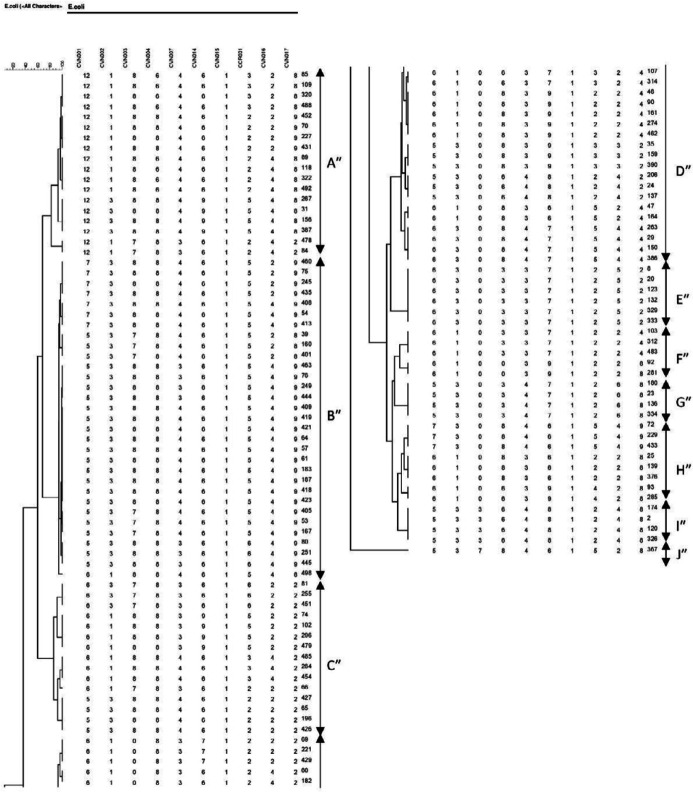
Clustering results of β -lactamase-producing *E. coli *using 10-loci MLVA

The β -lactamase-producing *E. coli* formed 4 major clusters by 10-loci designated A″ to J″. 10- loci MLVA-typing divided the β-lactamase-producing *E. coli* isolates into 36 distinct genotypes and 10 clusters (Figure 3). 

***Comparison of MLVA and PFGE profiles***

Analysis of the composition of the seven VNTR (variable number tandem repeat) loci of the strains collection revealed that the number of alleles ranged from one for CVN015 to 5 for CVN001. The diversity indices of the VNTR loci, measured by Simpson’s diversity index, showed an average diversity of 0.54. Analysis of the composition of the ten VNTR loci of the strains collection revealed that the number of alleles ranged from one for CVN015 to 5 for CVN001, CVN016, and CCR001. The diversity indices of the VNTR loci, measured by Simpson’s diversity index, showed an average diversity of 0.48. The lowest diversity index was found for CVN015, as 100% of the isolates had the same allele at this locus, and the highest diversity index was found for CVN001 in 7-loci MLVA and CVN001, CVN016, and CCR001 in 10-loci MLVA. PFGE analysis identified 49 distinct PFGE patterns among the isolates. 

PFGE showed to be more discriminatory than MLVA; the discriminatory power of 7-loci MLVA was calculated as 0.54 (Simpson’s diversity index) with a 95% confidence interval, while the discriminatory power of 10-loci MLVA was estimated at 0.48 with a 95% confidence interval. The discriminatory power of PFGE showed a value of 0.87 with a 95% confidence interval (Table 1). The discordance between the methods was high.

**Table 1:** Primer sequences for subtyping of beta-lactamase-producing E.coli isolates

**Target locus**		**Primer sequence**	**Reference**
***CVN001***	F	AACCGGCTGGGGCGAATCC	[11]
	R	GGCGGCGGTGTCAGCAAATC	
***CVN002***	F	AACCGTTATGAARGRAAGTCCT	
	R	TCGCCCAGTAAGTATGAAATC	
***CVN003***	F	AAAAATCCGGATGAGWTGGTC	
	R	TTGCGTTGTCAGTAATTTGTTCAG	
***CVN004***	F	MGCTGCGGCRCTGAAGAAGA	
	R	CCCGGCAGGCGAAGCATTGT	
***CVN007***	F	ACCGTGGCTCCAGYTGATTTC	
	R	ACCAGTGTTGCGCCCAGTGTC	
***CVN014***	F	TCCCCGCAATCAGCAAMACAAAGA	
	R	GCAGCRGGGACAACGGAAGC	
***CVN015***	F	TAGGCATAGCGCACAGACAGATAA	
	R	GTACCGCCGAACTTCAACACTC	
***CVN016***	F	GCTGCAGGAGAATGGGATGGTTTT	[12]
	R	GGTGAGGTGTCCGAGTGGCTGAAG	
***CVN017***	F	GCAATCACCGCCGCAATCTGTT	
	R	CGCCGCCGAAGCAAATCTC	
***CCR001***	F	CTCAGGGAAAAGGGAAGACACTAC	
	R	TTGCACTGAACACCGAATACG	

MLVA: multiple-locus variable-number tandem repeat analysis

PFGE: pulsed-field gel electrophoresis

**Table 2:** Comparison of Simpson's index of diversity for various MLVA and PFGE

**Typing method**	**Simpson's index of diversity**
**7-loci MLVA**	0.54
**10-loci MLVA**	0.48
**PFGE**	0.87

PFGE: pulsed-field gel electrophoresis; MLVA: multiple-locus variable-number tandem repeat analysis

MLVA: multiple-locus variable-number tandem repeat analysis

MLVA: multiple-locus variable-number tandem repeat analysis

## Discussion

Epidemiological studies of bacterial isolates can be performed by phenotypic and genotypic approaches. The discriminatory power of phenotypic methods is less than genotypic approaches. This subject can be explained by the gradual replacement of phenotypic methods by genotypic techniques within the past two decades (22-24).

A large number of genetic typing methods with a high degree of discriminatory power are utilized for molecular typing of bacterial isolates. PFGE is one of the most frequently used molecular genetic methods. PFGE is considered a gold standard method due to its excellent discriminatory power and high epidemiological concordance; however, the method remains relatively expensive and time-consuming (25, 26). Multilocus variable-number tandem repeat analysis (MLVA) is based on a set of polymorphic tandem repeat loci of bacteria and can be successfully used for epidemiological studies. MLVA has ideal criteria for typing, such as being easy to perform, rapid, possessing a high discriminatory power, and having good reproducibility (26, 27). 

The rapidly expanding β-lactamase-producing *E. coli* isolates and the continuous transmission of strains from the community to hospital settings (28-30) make the surveillance of hospital-acquired infections and attention to potential outbreaks a demanding challenge. In order to select the appropriate typing method according to the existing laboratory conditions, we evaluated and compared the use of 7-loci MLVA, 10-loci MLVA, and PFGE (2, 19) on β -lactamase-producing *E. coli *isolates. In this study, we compared the efficiency of PFGE and MLVA based on the previously reported 7-loci and 10-loci (31, 32) for typing of 130 ESBL-producing *E. coli* isolated from Iranian patients with UTI. There are different genotyping methods to investigate the epidemiology of *E. coli *(33) but PFGE has been the golden standard method for several years due to its discriminatory power (6, 34). In contrast to PFGE, MLVA is rapid, cost-effective, and easy to interpret and is a PCR-based typing method(35, 36).

The geographical region and the duration of the research can also have an effect on the population of bacterial isolates (37, 38), so an appropriate and standard method should be selected for molecular typing of the microorganisms in the intralaboratories network.

Lindstedt *et al*. in 2007 were the first to describe an MLVA scheme for *E. coli* based on 7 different VNTR loci (CVN001, CVN002, CVN003, CVN004, CVN007, CVN014, and CVN015) (19). Løbersli *et al.* 2012 described a 10-loci scheme for *E. coli *using three more VNTRs (CCR001, CVN016, and CVN017) (20). These additional loci improved the discriminatory capacity, as reported in Kim’s and Boxourd’s studies (39, 40). In their studies, 10-loci MLVA has better discriminatory power than 7-loci MLVA. Our results showed that some loci have the same number of repeats. Such repeats can change the basis of geographical regions; therefore, we can use selective geographical region-based VNTR loci. In our study, calculation of the diversity indices for the MLVA loci showed that CVN001, CVN016, and CCR001 were the most polymorphic with 5 different alleles. CVN015 was the least variable between our strains with one allele. Obtaining such a low diversity for CVN015 suggests that this should be replaced with a more polymorphic VNTR. According to Bustamante’s suggestion, 10-loci MLVA can be used in order to achieve a greater discrimination power (41). Observed results for 7-loci and 10-loci MLVA methods showed that polymorphic VNTRs must be selected precisely.

If the MLVA method is used for molecular typing of bacterial isolates, it is possible that one or two loci of the typed strains are failed. This may have been due to the fact that the corresponding locus was missing. This event occurs due to long VNTRs (42, 43). In Nakamura’s study, MLVA has been a suitable suggestion for short-range epidemiological studies owing to its ability to recognize closely related strains (44). 

In this study, the PFGE method was performed by one restriction enzyme as XbaI. The discriminatory power of the PFGE method was more than 10-loci and 7-loci MLVA. PFGE demonstrated the clonal spread of the strains in the hospitals showing the vertical transmission of antimicrobial resistance and poor infection control strategies in these healthcare settings. Results of 7-loci were very close to those of 10-loci MLVA.

In the PFGE method, the comparison of bacterial isolates was performed based on all fragments under comparison with the same position on the gel, so once we know their position, we can examine their genetic identity and determine if they have one source or they are clones of the same strain. According to PFGE patterns, gene transfer in isolates can take place horizontally or vertically (45). It has been proven that PFGE has more discriminatory power than MLVA methods (46). Our findings suggest that PFGE has more discriminatory power than MLVA. The choice of the restriction enzyme and conditions for electrophoresis need to be optimized and they depend on the bacterial species (47). Additional restriction enzymes can improve the value of the PFGE method for differentiating highly homogeneous *E. coli* strains (48, 49). Genome digestion with a second or a third enzyme mostly improves the discriminatory power of PFGE (50). 

Zeibel *et al*. in 2017 have investigated the combined typing methods for improving the discriminatory power (51). They have found combining the typing methods can improve the discriminatory power, for example, combining the MLVA subtype with phage typing (PT) increased the discrimination of subtypes as well as the diversity index (DI) even though this DI was still significantly lower than the DI of PFGE plus PT. In contrast, combining PFGE and MLVA subtypes increased the number of subtypes to 64 and generated a DI close to 1. Although more subtypes were identified by PFGE than by MLVA, some PFGE subtypes and PTs were further split by MLVA, thus supporting that MLVA may prove useful by providing additional discrimination to these existing typing methods (51).

Following the results obtained in this study, it is suggested to investigate molecular typing techniques in combination, such as MLVA and phage typing, for typing of bacterial isolates.

## Conclusion

Our study showed that PFGE is more discriminatory than MVLA. Genotyping of *E. coli* is essential for monitoring the spread of β -lactamase-producing strains, implementation of suitable infection control strategies, and general epidemiology. Such purposes need development of a high-resolution genotyping tool with a fixed scheme. MLVA is a PCR-based method and is valuable as a screening tool for epidemiological study of an outbreak, but optimizing polymorphic VNTR loci is vital to improving the discriminatory power of the method.

## Ethics

This study and all procedures performed were approved by the Ethics Committee of Islamic Azad University of IRAN (registration number IR.IAU.PS.REC.1397.306).

## Data Availability

In this study, we tried to investigate the most common technique for subtyping AmpC-producing *E. coli* and select an effective technique. All data are available upon request.
